# Investigation of the mechanism of dural arteriovenous fistula formation induced by high intracranial venous pressure in a rabbit model

**DOI:** 10.1186/1471-2202-15-101

**Published:** 2014-08-27

**Authors:** Shou-Sen Wang, Chang-Hua Li, Xiao-Jun Zhang, Ru-Mi Wang

**Affiliations:** Department of Neurosurgery, Fuzhou General Hospital, Fujian Medical University, 156 Road Xi’erhuanbei, Fuzhou, 350025 Fujian China

**Keywords:** Dural arteriovenous fistula, Rabbit model, High intracranial venous pressure, Hypoxia inducible factor-1α, Vascular endothelial growth factor

## Abstract

**Background:**

The causes of dural arteriovenous fistula have not been clearly defined. The aim of this study was to investigate the mechanism of dural arteriovenous fistula formation induced by high intracranial venous pressure using a rabbit model.

**Results:**

By using rabbit model, dural arteriovenous fistula formation induced by high intracranial venous pressure could be produced by end-to-end and end-to-side anastomosis of the right side common carotid artery with the posterior facial vein plus ligation of the contralateral external jugular vein. As compared the post arteriovenous fistula formation among 1 week, 2 weeks, 3 weeks, and 90 days, the expression level of vascular endothelial growth factor in the 1- and 2-weeks groups was significantly higher compared with the control group, 3 weeks and 90 days groups (p ≤0.002). There was significantly higher hypoxia inducible factor-1α expression in the one week group compared with the control, 2 weeks, 3 weeks, and 90 days groups (p ≤0.002). The results of Western blotting showed that vascular endothelial growth factor expression level was highest in the 1 week group. The expression level of vascular endothelial growth factor was significantly different between all groups.

**Conclusions:**

The results of the experiments in our rabbit model indicate that high intracranial venous pressure is a key for dural arteriovenous fistula formation. Cerebral ischemia caused by lack of cerebral perfusion pressure plays a key role in the process that leads from high intracranial venous pressure to increased hypoxia inducible factor-1α expression and then increased vascular endothelial growth factor expression.

**Electronic supplementary material:**

The online version of this article (doi:10.1186/1471-2202-15-101) contains supplementary material, which is available to authorized users.

## Background

A dural arteriovenous fistula (DAVF) is an abnormal connection between dural arteries and dural veins or a venous sinus or cortical veins. It is a type of intracranial vascular malformation. Dural arteriovenous fistulas account for about 10-15% of all intracranial vascular malformations, 6% of supratentorial arteriovenous malformations and 35% of infratentorial arteriovenous malformations [1]. A dural arteriovenous fistula may occur at any site, but these vascular malformations most commonly occur in the cavernous sinus, transverse sinus, sigmoid sinus and superior sagittal sinus (SSS). The main form of treatment for DAVF is endovascular embolization [2]. However, Cha et al. reported the outcomes of 43 DAVF patients treated with transvenous embolization or transarterial embolization and concluded that a new approach to treatment of DAVF is needed [2].

The causes of DAVF have not been clearly defined. It is thought that there are both congenital and acquired causes. There is a view that DAVF is an intracranial arteriovenous malformation and a congenital disease caused by dural vascular abnormalities [3]. However, few DAVF cases have involved infants and evidence supporting this view is lacking. There have been many clinical trials that have shown that DAVF formation may be caused by brain trauma, venous sinus inflammation, venous sinus thrombosis formation, intracranial tumors, brain surgery, hypercoagulable states, blood molecular abnormalities, etc., which supports the view that DAVF is an acquired vascular disease. Among the reasons why it is believed that DAVF is acquired is that the connection between DAVF and venous sinus thrombosis formation is relatively close. Many investigators have established rat models of high venous sinus pressure using the common carotid artery-external jugular vein (CCA-EJV) anastomosis method and found that 1) the high venous sinus pressure may induce DAVF; 2) DAVF disappeared automatically after removing the high venous sinus pressure; 3) The venous pressure in the high-pressure groups increased significantly after surgery [4], which became normal 28 days after surgery; 4) The venous sinus thrombosis was a risk factor of the high venous sinus pressure [5]. The key point in favor of DAVF formation being due to acquired causes is the presence of high intracranial venous sinus pressure. There are two theoretical explanations: one is the opening of “physiological arteriovenous anastomoses,” and the other is “vascular endothelial growth factor-induced dural angiogenesis.”

Lawton et al. established a rat model of high venous sinus pressure using end-to-end anastomosis (EEA) between the CCA and the EJV [6]. They further transplanted the rat dura to the cornea of rabbits and observed obvious angiogenesis on the cornea. This result suggests that high venous sinus pressure not only can promote expression of vascular endothelial growth factor (VEGF), but also can induce DAVF formation. The researchers proposed that high intracranial pressure is an important factor in the pathogenesis of DAVF, and that there are various causes of vein occlusion and subsequent obstruction of the venous circulation, which increases the intracranial venous pressure and further induces a series of reactions. They concluded that DAVF formation is a result of aberrant angiogenesis.

There is strong evidence that factors associated with angiogenesis are involved in the formation of DAVF. The aim of this study was to investigate in more detail the mechanism of DAVF formation induced by high intracranial venous pressure using a rabbit model. This study is the first to develop a rabbit model using CCA-PFV anastomosis to generate a high intracranial venous pressure model and successfully induce DAVF formation and may be the first to find that hypoxia inducible factor-1α (HIF-1α) and vascular VEGF are expressed in sequential order in the rabbit model.

## Methods

### Materials

#### Experimental animals

This study was approved by the Institutional Review Board of Fuzhou General Hospital. One hundread Japanese long-ear male rabbits (weight: 2.0-2.5 kg) were provided by the Shanghai SLAC Laboratory Animal Co.; the license number is SCXK (Shanghai) 2010-0005. The rabbits were reared in the Department of Comparative Medicine, Fuzhou General Hospital in accordance with guide for the care and use of laboratory animals.

#### Anesthesia

Anesthesia was induced by injecting 1% sodium pentobarbital (25 mg/kg) into the ear vein (EV) for procedures including arteriovenous anastomosis, placement of a catheter in the carotid artery, harvesting the specimens, etc. One percent sodium pentobarbital was injected through the catheter into the carotid artery for cerebrovascular angiography.

### Methods

#### Preparation of animal model

**Grouping of experimental animals** Fifty male Japanese long-ear rabbits were randomly divided into five groups, A-E. In group A (control group) a sham operation was performed (n = 10). In group B, right-side CCA-posterior facial vein (PFV) EEA was carried out (n = 10). In group C, right-side CCA-PFV EEA plus left-side EJV ligation was performed (n = 10). In group D, right-side CCA-PFV end-to-side anastomosis (ESA) was carried out (n = 10). In group E, right-sided CCA-PFV ESA together with left-sided EJV ligation was performed (n = 10). At 7, 14 and 90 days after surgery, two rabbits were randomly selected from each group and the number of dural microvessels was counted after ink perfusion. At 90 days after surgery, four rabbits from each group were selected for digital subtraction angiography (DSA) and the status of DAVF formation was observed.

Another fifty male Japanese long-ear rabbits were randomly divided into five groups, A-E. In group A (control group) a sham operation was performed (n = 10). In group B-E (total n- = 40), righht-side CCA-PFV EEA plus left-side EJV ligation was performed. In group B, C, D, and E (n = 10/each group), specimens were taken at one week, two weeks, three weeks, and 90 days after surgery, respectively, for further immunohistochemistry (n = 6 for each group) and western blotting (n = 4 for each group) analyses.

**Preparation of model** Animals were fasted for 10 h before surgery without water restriction. One percent sodium pentobarbital (25 mg/kg) was injected into the left EV. After anesthesia, the rabbit was fixed on the operating table. After the neck area was sterilized with Anerdian, a skin incision was made in the neck. The following procedures were carried out under a microscope.

**Figure 1:**
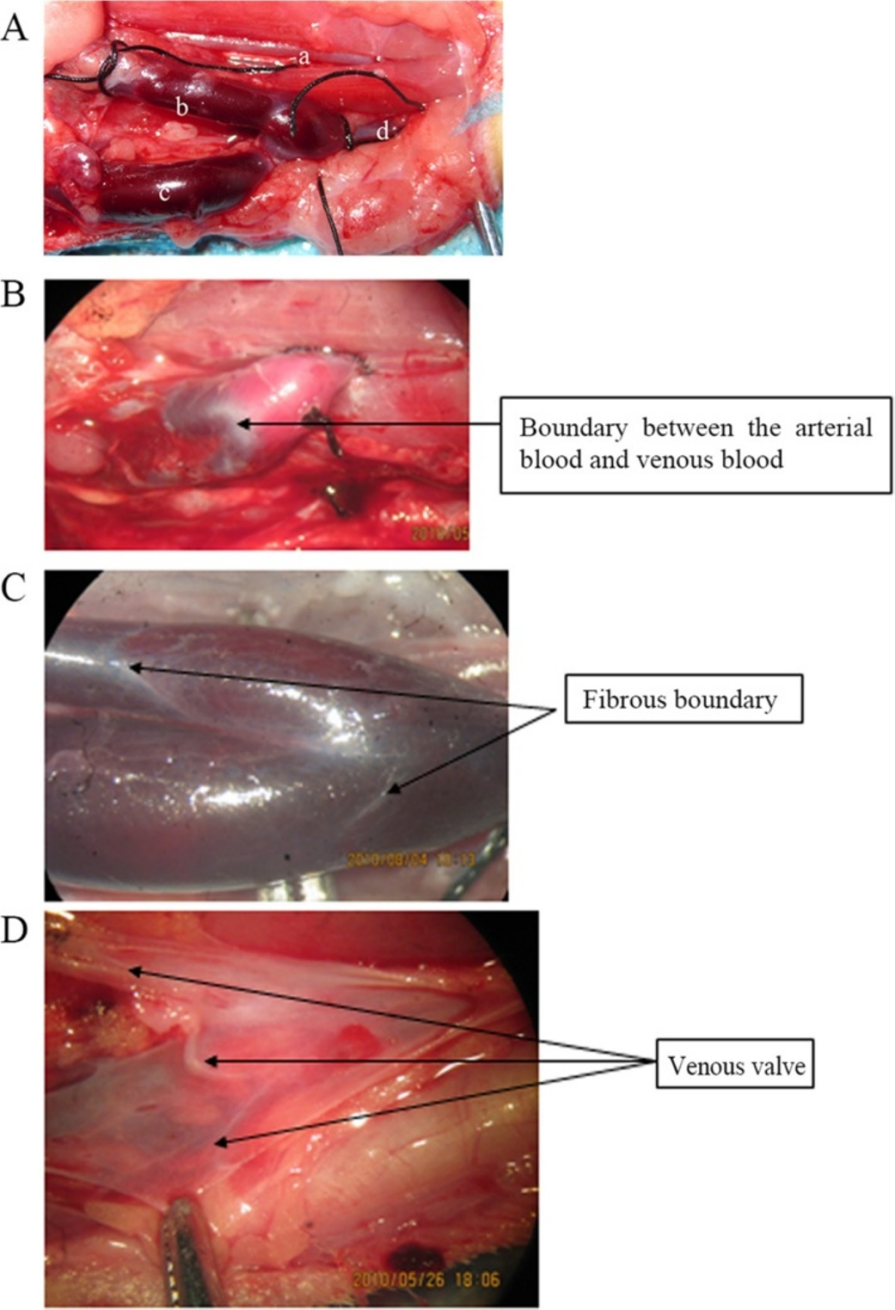
**Anatomical view of common carotid artery-external jugular vein area. A**. The rabbit was placed in the supine position, a midline neck incision was made and structures in the left neck were exposed. a. Common carotid artery; b. Anterior facial vein; c. Posterior facial vein; d. External jugular vein. **B**. In the preliminary experiment, a significant boundary between the arterial blood and venous blood was observed after end-to-side anastomosis between the CCA and EJV. **C**. Before the anterior facial vein and the posterior facial vein join to form the external jugular vein, there is a significant fibrous boundary. **D**. Longitudinal incision of the veins shows that there is one valve in the junction of the anterior facial vein and the external jugular vein and one valve in the junction of the posterior facial vein and the external jugular vein. Both valves are pointing cephalically to prevent the reflux of venous blood from the chest and abdomen into the head. Without these valves, the venous blood reflux into the head may cause obstruction of the venous drainage from the head and further induce intracranial ischemia and hypoxia (similar to the situation of hanging upside down).

1. Group A: The bilateral external jugular veins were dissected subcutaneously. The proximal end was 1 cm inferior to the junction of the anterior and posterior facial veins and the EJV. The distal end was 1 cm superior to the junction of the anterior and posterior facial veins and the EJV. The right carotid triangle was exposed and about 2 cm of the CCA was dissected bluntly (Figure 1). Next, 24# intravenous (IV) catheters were punctured into the posterior facial vein (PFV) and the CCA respectively and connected to an invasive pressure measuring instrument to measure the pressures of the PFV and the CCA. The incision was closed after topical application of a small amount of penicillin.
2. Group B: The bilateral external jugular veins and the right CCA were dissected (the lengths were equal to those of the sham operation group). The normal artery pressure and the PFV pressure were measured. The proximal end of the right EJV was ligated (0.5 cm inferior to the junction of the anterior and posterior facial veins and the EJV). The distal end of the anterior facial vein (AFV) was also ligated (1 cm superior to the junction of the AFV and the EJV). The PFV was clipped with a vascular clip (1 cm superior to the junction of the PFV and the EJV) and cut off about 3 cm superior to the junction of the PFV and the EJV (across the vein valve). The proximal end of the CCA was clipped, the distal end was ligated and cut off, and the lumen of the blood vessel was washed with 1 mg/ml heparin/saline solution. The stumps of the CCA and the PFA were stained with methylene blue and then washed with 1 mg/ml heparin/saline solution. End-to-end anastomosis between the CCA and the PFV was carried out using 9-0 suture line. The anastomotic patency was verified after anastomosis. The PFV pressure was measured after anastomosis, and the incision was closed after topical application of a small amount of penicillin.
3. Group C: The right CCA, EJV and the left EJV were dissected. The left EJV was ligated using 4-0 suture line. Other procedures were the same as in group B.
4. Group D: The bilateral external jugular veins and right CCA were dissected (the length of dissection was the same as in the sham operation group). The normal artery pressure and the PFV pressure were measured. The proximal end of the right EJV and the distal end of the AFV were ligated. The PFV was clipped with a clip and cut off at a point 3 mm distal to the junction of the PFV and the EJV. The lumen of blood vessel was washed with 1 mg/ml heparin/saline solution. Two vascular clips were used to clip the CCA, and the distance between two clips was about 1.5 cm. An incision was made along the vessel wall using a microsissor, and the length was 1.5 times the length of the vascular diameter. The lumen of the blood vessel was washed with 1 mg/ml heparin/saline solution immediately. The incision in the CCA and the stump of the PFV were stained with methylene blue and washed with 1 mg/ml heparin/saline solution. End-to-side anastomosis between the common carotid artery and the PFV was carried out using 9-0 suture line. The anastomotic patency was verified after anastomosis. The PFV pressure was measured after anastomosis, and the incision was closed after topical application of a small amount of penicillin.
5. Group E: The right CCA, EJV and the left EJV were dissected. The left EJV was ligated using 4-0 suture line. Other procedures were the same as in group D.

Additional file 1: Figure S1 represented schematic drawings of groups A-E, which illustrates the vessels that were occluded and anastomosed as well as the collaterals.

#### Measurement of pressures

Dissection of the CCA and measurement of the CCA pressure were carried out in all 50 rabbits in the five groups (A-E).

**Figure 2:**
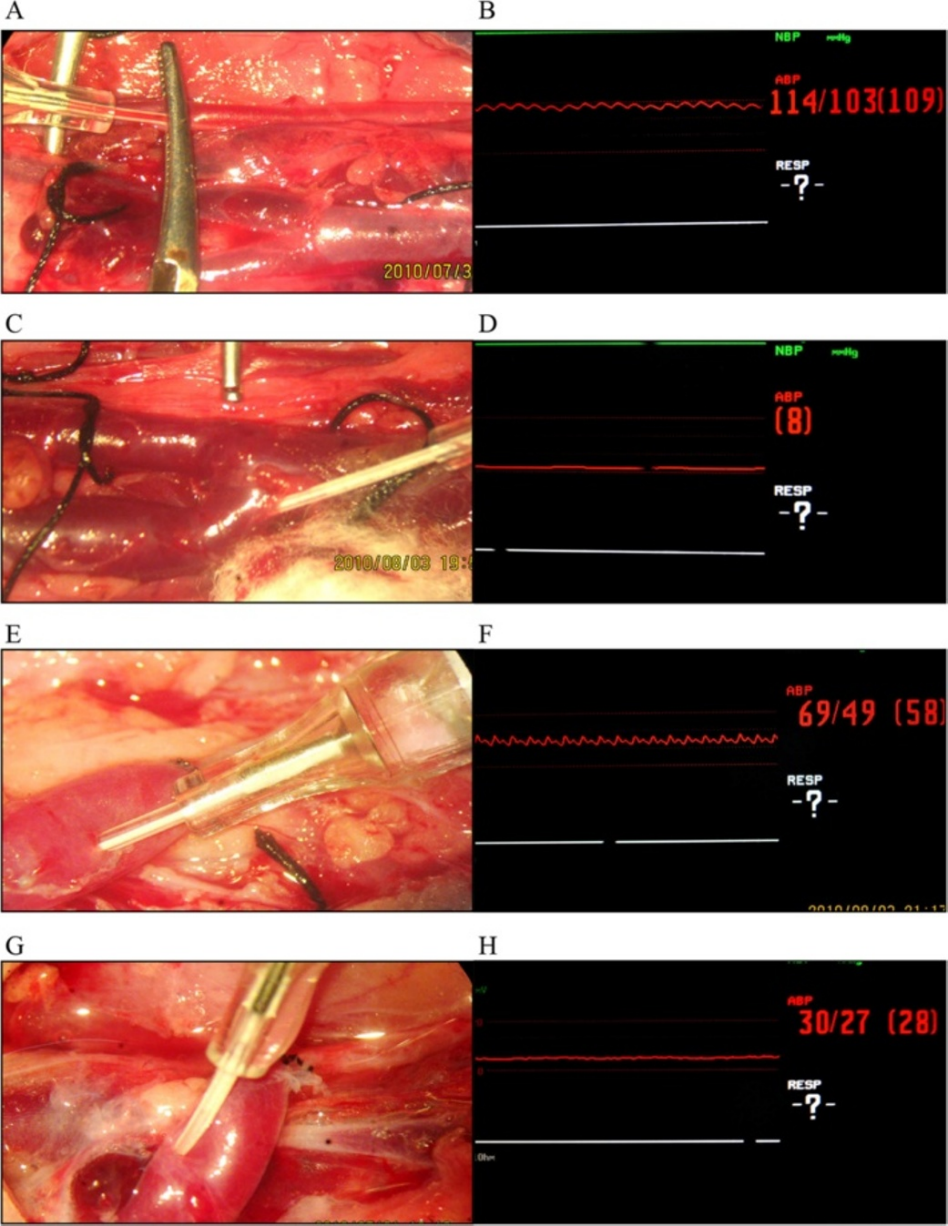
**Measurement of the pressure of the right cervical blood vessels (left is the cephalic side). A**. Fixation method and measurement of the common carotid artery pressure: An IV catheter was inserted caudally into the common carotid artery and fixed with a vascular clip. **B**. The normal artery pressure. **C**. Measurement of the posterior facial vein pressure: An IV catheter was inserted cephalically into the posterior facial vein and no special fixation was carried out. **D**. The normal posterior facial vein pressure. **E**. Measurement of the posterior facial vein pressure after end-to-end anastomosis (EEA): An IV catheter was inserted cephalically into the posterior facial vein and fixed. **F**. The posterior facial vein pressure after EEA showed an arterial pressure-like change. **G**. Measurement of the posterior facial vein pressure after end-to-side anastomosis: An IV catheter was inserted cephalically into the posterior facial vein and fixed. **H**. The posterior facial vein pressure after end-to-side anastomosis shows an arterial pressure-like change.

**Measurement of blood pressure in the common carotid artery** The right CCA was dissected about 2 cm and a 24# IV catheter was inserted caudally, fixed with a vascular clip and connected with an invasive pressure measuring instrument (Figure 2A). The pressure was adjusted to zero at the heart level. After the reading became stable, photographing and recording were carried out (Figure 2B).

**Measurement of posterior facial vein pressure** Dissection of the PFV and measurement of the PFV pressure were carried out for all 50 rabbits in the five groups (A-E). The total length of dissection of the right EJV, and anterior and posterior facial veins was about 2 cm. A 24# IV catheter was inserted cephalically through the vein valve into the PFV and connected with an invasive pressure measuring instrument after fixation (Figure 2C). The pressure was adjusted to zero at the heart level. After the reading became stable, photographing and recording were carried out (Figure 2D).

**Measurement of posterior facial vein pressure after anastomosis** For the 40 rabbits in groups B-E, the anastomotic patency was verified after CCA-PFV anastomosis and before sacrifice and the PFV pressure was measured. At the PFV side of anastomosis, a 24# IV catheter was inserted cephalically and connected with an invasive pressure measuring instrument after fixation (Figure 2E and 2G). The pressure was adjusted to zero at the heart level. After the reading became stable, photographing and recording were carried out (Figure 2F and 2H).

### Ink perfusion

Two rabbits were selected from group A and at 7, 14, and 90 days after CCA-PFV anastomosis in groups B-E for ink perfusion in the head and neck. Microvessel density of the harvested dura was counted.

### DSA examination

At 90 days after surgical intervention, four rabbits were selected from the five groups for DSA examination of the head and neck. Detailed procedures were as follows:

**Catheter placement in the carotid artery** After being anesthetized by EV injection of 1% pentobarbital sodium (1% 25 mg/kg), the rabbit was placed in the supine position and fixed on the operating table. Dissection was carried out along the original neck incision. The site of the right arteriovenous anastomosis was carefully dissected. There were numerous new veins formed around the site. After verifying the anastomotic patency, the posterior facial vein pressure was measured. The site of anastomosis was ligated and fixed. The left CCA was dissected and a 24G IV catheter was placed in it. The catheter was filled with 1 mg/ml heparin and fixed securely. The neck incision was closed.

**Obtaining of DSA images** After being anesthetized by CCA injection of 1% pentobarbital sodium, the rabbit was placed in the supine position and fixed on the DSA table. After adjusting the machine position, iohexol-300 was injected through the catheter placed in the CCA for angiography (2 ml/s, 3 ml). Anteroposterior radiographs were then taken.

## Immunohistochemistry

Specimens of occipital cortex and dura were collected from the control, one week, two weeks, three weeks, and 90 days groups, respectively (n = 6 each group) for HIF-1α and VEGF immunohistochemistry. The occipital cortex was chosen because it is the area first affected by the impact of anastomosis generating high intracranial venous pressure and it is conveniently located for conducting experiments. Immunohistochemistry was performed in 5-mm sections of tissues that were formalin-fixed and paraffin-embedded. Reference sections were stained with hematoxylin and eosin (HE). The tissue sections were incubated with the following primary antibodies: VEGF (C-1) (1:100; Santa Cruz, Santa Cruz, CA, USA), HIF-1α (1:200; H-206; Santa Cruz, Santa Cruz, CA, USA) at 4°C for overnight. Then incubated with a horseradish peroxidase-conjugated mouse anti-rabbit secondary antibody (1:1000). Immunohistochemical analysis was performed using the s 3,3’-Diaminobenzidine (DAB) (Sigma-Aldrich, St Louis, MO, USA) method. All specimens were analyzed under inverted microscope and photographic systems Olympus BX51 (Olympus, Tokyo, Japan). The visual fields with 100× and 200× magnification were 1.8-2 mm^2^ and 0.9-1 mm^2^, respectively. The percentage was expressed as stained “positive” cell divided by the total number of cells under the same magnifications (i.e., 100× or 200×) of the same visual areas. The criteria used to assign the numerical values to expression levels were the following: - = 0%, + = >0%-25%, ++ = 26%-50%, +++ = 51%-75%, ++++ = >75%.

## Western blotting

Specimens of occipital cortex and dura were collected from the control, one week, two weeks, three weeks, and 90 days groups, respectively (n = 4 each group) for Western blotting. Specimens were lysed in RIPA buffer (Millipore, Billerica, MA, USA), and the concentration of the protein extracts was determined by the Bradford assay (Bio-Rad Protein Assay Kit 500-0201; Life Science, Hercules, CA, USA). A total of 40 μg total protein was separated by 10% SDS-PAGE and then transferred onto a polyvinylidene fluoride (PVDF) membrane, which was incubated in blocking buffer of 5% non-fat milk in TBS containing 0.1% Tween-20. The membrane was then probed with a primary antibodies specific for VEGF (C-1) (1:200; Santa Cruz, Santa Cruz, CA, USA ), HIF-1α (1:200; H-206; Santa Cruz, Santa Cruz, CA, USA), and the loading control, α-tubulin (52 kDa, 1:3000; 2125; Cell Signaling Technology) at 4°C for overnight. The membranes were next incubated with a horseradish peroxidase-conjugated mouse anti-rabbit secondary antibody (1:1000). Immunoreactive bands were visualized using enhanced chemiluminescent HRP Substrate (Millipore). Results represent three independent experiments. Densitometric analysis was performed by GE image scanner Quantity One software (Fairfield, CT, USA).

## Statistical analysis

Continuous data were expressed as the mean with standard deviation. Categorical data were expressed by the number in each group. The comparisons of the animals’ weight, arterial blood pressure, and venous blood pressure among the five groups of rabbits that underwent various operations as well as the comparisons of the levels of expressions of VEGF and HIF-1α by Western blotting among the five groups at baseline, 1 week, 2 weeks, 3 weeks, and 90 days were analyzed, respectively, by one-way ANOVA (analysis of variance) together with Bonferroni post-hoc tests. Changes of venous blood pressure from baseline to post-operation were compared and analyzed by the paired t-test. The associations of anastomosis patency versus operation groups were analyzed by the Fisher’s exact test. The comparisons of the ordinal data (VEGF and HIF-1α expression levels by immunohistochemistry) among all five groups and post-hoc test for each two groups were performed by the non-parametric Kruskal-Wallis test and Mann-Whitney test with Bonferroni correction. All statistical assessments were two-tailed and evaluated at the 0.05 level of significant difference. Statistical analyses were performed using SPSS 15.0 statistics software (SPSS Inc, Chicago, IL, USA).

## Results

The comparisons of baseline weight and blood pressure among the five groups (A-E) are summarized in Table 1. There were no significant differences among the five groups.

**Table 1:** **Summary of the peri-operative characteristics of the animals in the five groups**

	A (n = 10)	B (n = 10)	C (n = 10)	D (n = 10)	E (n = 10)	P-value
**Baseline**						
Weight (kg)	2.23 (0.19)	2.29 (0.19)	2.23 (0.20)	2.30 (0.16)	2.17 (0.16)	0.508
Systolic artery BP (mmHg)	109.60 (4.84)	112.40 (12.52)	108.30 (8.42)	108.20 (11.58)	105.70 (12.39)	0.698
Diastolic artery BP (mmHg)	98.80 (7.58)	101.90 (10.58)	96.60 (12.95)	99.40 (10.20)	92.88 (7.57)	0.417
Venous BP (mmHg)	5.90 (1.45)	5.90 (2.02)	7.00 (2.21)	6.40 (1.26)	5.80 (2.15)	0.572
**Immediately after operation**						
Frequency of anastomosis patency	10/10	8/10	9/10	6/10	5/10	0.052
Systolic venous BP (mmHg)	6.20 (1.69)	49.50 (14.77)*^a^	54.70 (15.23)*^a^	47.90 (16.14)*^a^	46.50 (19.88)*^a^	<0.001
Diastolic venous BP (mmHg)		42.60 (13.08)*^a^	45.00 (12.07)*^a^	42.50 (15.06)*^a^	41.10 (18.72)*^a^	<0.001
**Before sacrifice**						
Venous BP (mmHg)	6.20 (1.87)	41.60 (12.71)*^a^	42.90 (10.73)*^a^	25.80 (13.45)*†^a^	26.00 (13.16)*†^a^	<0.001

Anastomosis patency is presented as number of each group; other data are presented as mean and standard deviation. A: sham group, B: end-to-end anastomosis group, C: end-to-end anastomosis + ligation of the contralateral EJV group, D: end-to-side anastomosis group, E: end-to-side anastomosis + ligation of the contralateral EJV group. *BP*: Blood pressure. *indicates a significant change compared to group A, †indicates a significant change compared to group B and group C. ^a^indicates a significant change compared to baseline level of venous BP.

In each group of ten rabbits six animals were used for ink perfusion and four were used for DSA examination/.

All animals in the groups B and C survived after surgery. Due to excessively high intracranial venous pressure, two rabbits in groups D and E died within 3 days after surgery. The rate of anastomotic patency was 80% in the group B, 90% in group C, 60% in group D and 50% in group E.

### Peri-operative blood pressure

No significant differences in the frequency of anastomosis patency were observed postoperatively among groups B-E. Post-operative venous blood pressure in the sham operation group (group A) did not change significantly compared to baseline. All four high venous pressure groups (groups B-E) had significantly increased venous blood pressure immediately after surgery and before sacrifice compared with baseline (p ≤ 0.001), and had significantly increased venous blood pressure immediately after surgery and before sacrifice compared to the control group (p < 0.001). The venous blood pressure of the animals in B and C groups was significantly higher compared with the animals in the D and E groups (41.6 and 42.9 mmHg vs. 25.8 and 26.0; p ≤ 0.003), respectively. However, there were no significant differences between groups B and C or between groups D and E groups (Table 1).

### Cerebral ink perfusion

**Figure 3:**
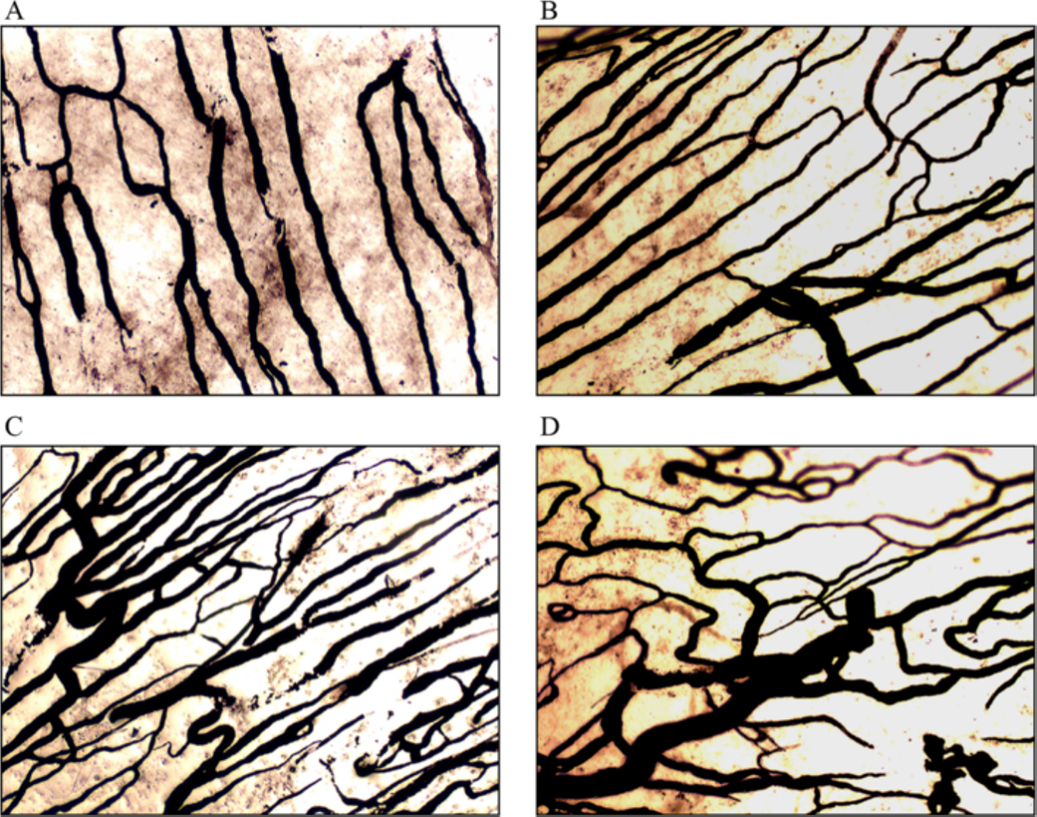
**Cerebral ink perfusion. A**. The dural blood vessels were parallel in the control group, there were few connections between the parallel blood vessels (100×). **B**. The dural blood vessels were parallel in the high-pressure groups 14 days after surgery, there were some connections between blood vessels and some enlarged veins (100×). **C**. The dural blood vessels were still parallel in the high-pressure groups 90 days after surgery, the blood-vessel enlargement was significant and the amount of connections between blood vessels increased significantly, and the diameters of the blood vessels for connection were relatively thin (100×). **D**. The artery was directly connected with the enlarged vein (200×).

Convex microvascular surface of dural capillaries comprised parasagittal sinuses 2 mm wide and 3 mm above the transverse sinuses were included for observations and comparisons. The dural capillaries were counted as numbers of microvessels/per mm^2^. The dural microvessel count of control group A was 12 ± 2/mm^2^ and increased slightly to 15 ± 2/mm^2^ at 14 days post-operation. After 90 days, the dural microvessel count were significantly increased among groups B, C, D, and E as 36 ± 4 mm^2^, 39 ± 5 mm^2^, 33 ± 3 mm^2^, and 35 ± 4 mm^2^, respectively. The results of cerebral ink perfusion are illustrated in Figure 3.

### DSA findings

**Figure 4:**
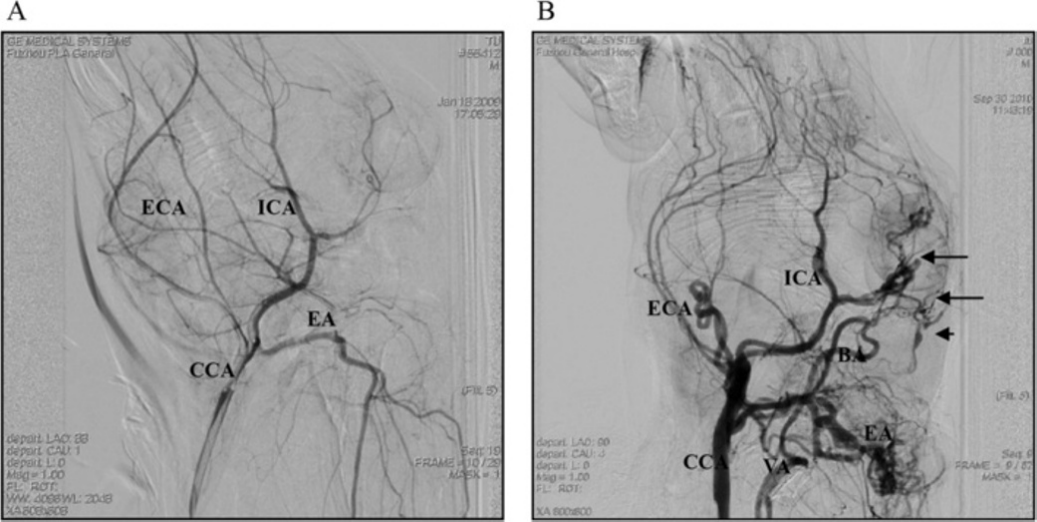
**DSA arterial phase.** **A**. Group A after 90 days, the left common carotid artery angiography showed normal radiographs in the arterial phase toward the common carotid artery (CCA), internal carotid artery (ICA), external carotid artery (ECA ), and ear artery (EA); **B**. group C after 90 days, the left common carotid artery angiography lateral arterial phase shows CCA, ECA, ICA, and EA toward tortuous, and visible abnormal development of the vertebral artery (VA), basilar artery (BA), DAVF, eye AVF, and the superior sagittal sinus (SSS).

**Figure 5:**
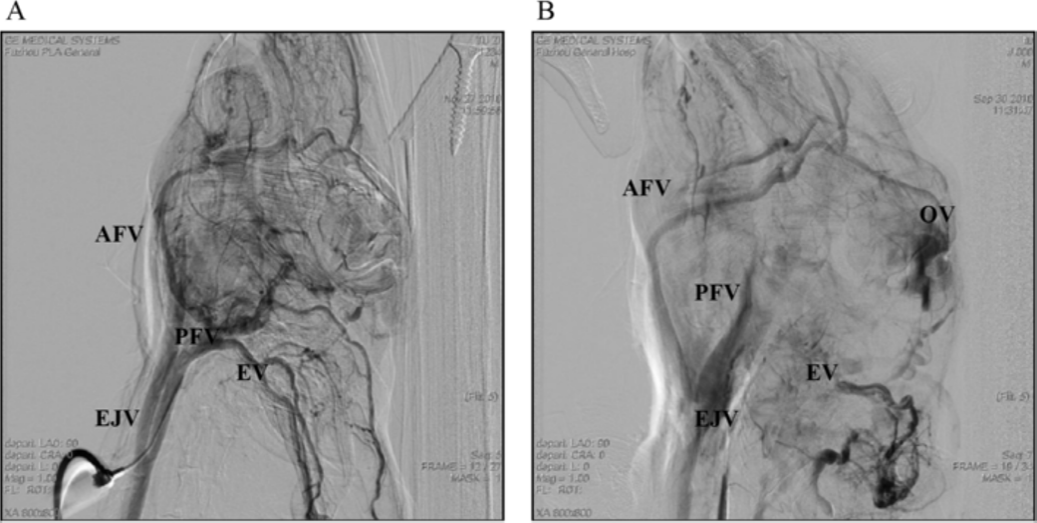
**DSA venous phase.** **A**. Group A after 90 days, the results from left common carotid artery angiography lateral veins at late stage had shown the surface normal to the posterior facial vein (PFV), before intravenous anterior facial vein (AFV), external jugular vein (EJV), and ear vein (EV) all run regularly; **B**. group C after 90 days, the left common carotid artery angiography lateral veins at late stage had shown ophthalmic vein (OV), intracranial venous and EV all had stranded flow.

DSA examinations were carried out for four rabbits from each group (A-E) 90 days after surgery. DSA images in the arterial phase are shown in Figure 4 and in the venous phase in Figure 5. In each figure an image from an animal in the control group is shown next to an image from an animal in group C. In group A, the blood vessels were unobstructed, no abnormal veins were observed, and the time for blood circulation was about 11-15 s. In groups B, C, D and E, the blood vessels were tortuous and DAVF and AVF in the ear and eye regions were observed. In some animals, two or more than two AVFs were observed. Twenty-two cases of AVF were observed in 16 rabbits. Among them, 7 cases were DAVFs, and therefore rate of DAVF formation was 43.75% (7/16). The DAVFs were mainly located in the SSS, cavernous sinus, transverse sinus. AVFs occurred at two sites in 6 animals and at three sites in one animal. DAVFs were drained via the SSS, transverse sinus and posterior facial vein; AVFs in the eye were drained via the anterior facial vein; AVFs in the ear were drained via the posterior facial vein; and the cervical AVF was also drained via the posterior facial vein. In the groups B, C, D and E, the circulation time was extended about 32-40 s (mean, 35 s). The circulation time was extended significantly in the venous phase while it was not obviously extended in the arterial phase (about 5 s). Circulation time was extended significantly in the high-pressure groups (equal to or more than 35 s) compared to group A; especially in the venous phase it was more obvious. It is suggested that the increased intracranial venous system pressure resulted in the obstruction of the blood drainage.

### VEGF and HIF-1α expression levels by immunohistochemistry

**Figure 6:**
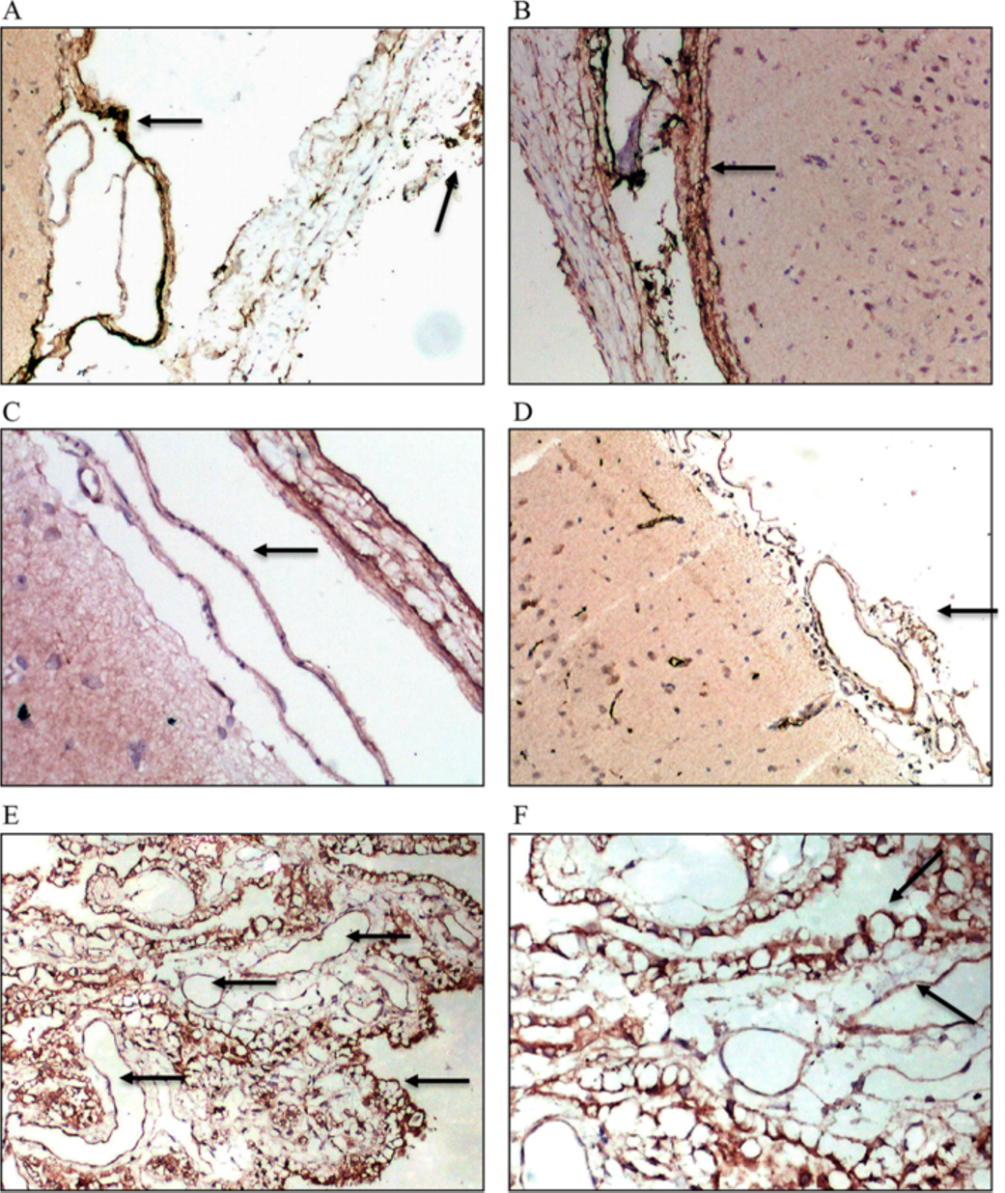
**Immunohistochemical VEGF staining.** Each of the five groups included six animals: **A**. One week group, occipital cortex and leptomeningeal vessels were VEGF (+), the dura mater was VEGF (±) (VEGF staining , 10 × 10); **B**. Two weeks group, occipital cortex soft meningeal blood vessels showed VEGF (+ +), the dura mater was VEGF (+) (VEGF staining, 10 × 10); **C**. three weeks group, occipital cortex and leptomeningeal vessels were VEGF (±), the dura mater was VEGF (+) (VEGF staining , 10 × 20); **D**. control group, occipital cortex and leptomeningeal vessels were VEGF (±) (VEGF staining , 10 × 10); **E**. 90 days group, dura was VEGF (+), and visible blood vessels increased in number (VEGF staining , 10 × 10); **F**. 90 day group, dura was VEGF (+), positive-stained granules are mainly located in vascular endothelium and vascular extracellular matrix and the number of visible blood vessels increased, lumen expansion (VEGF staining , 10 × 20).

**Figure 7:**
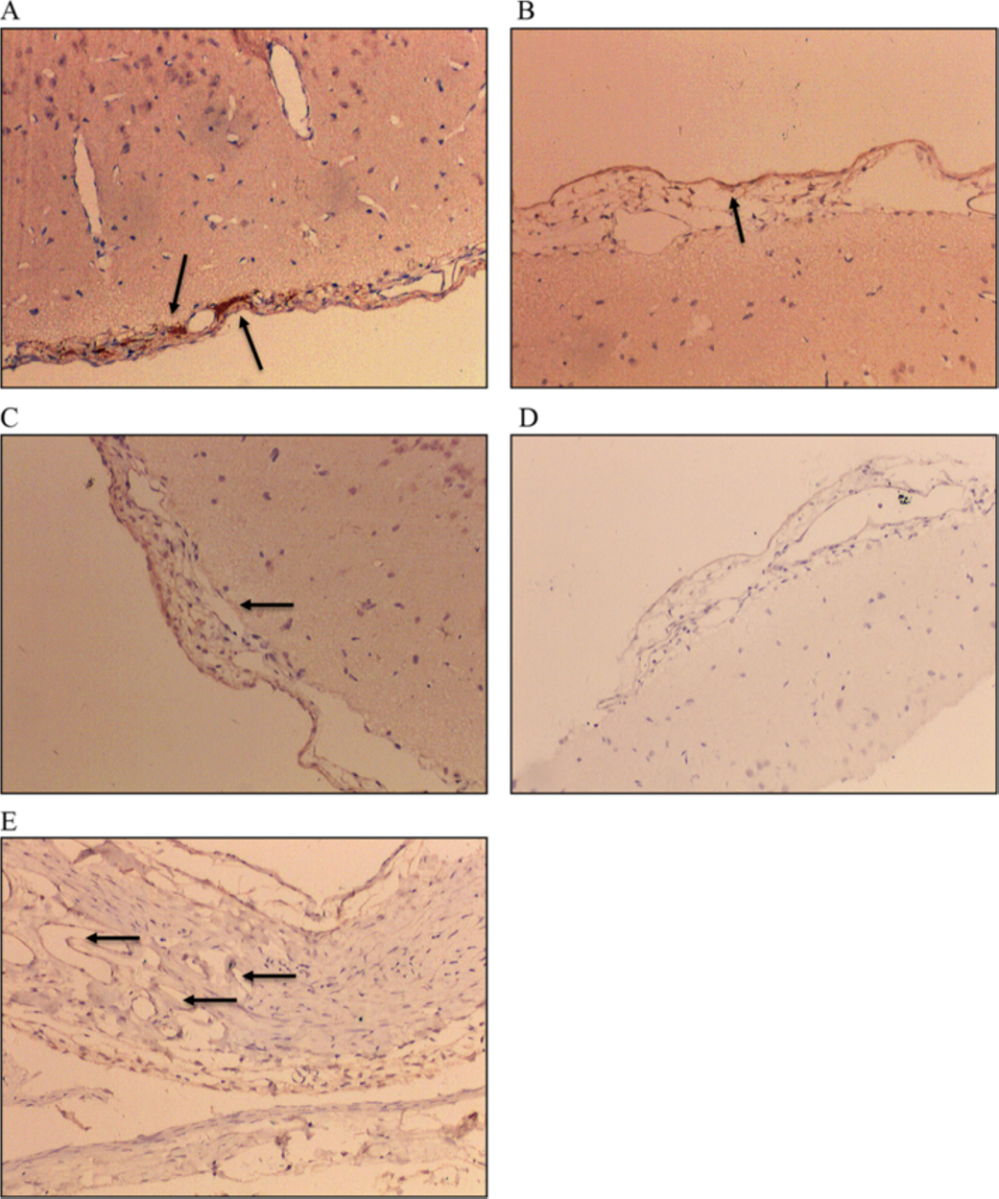
**HIF-1α immunohistochemical staining.** Each of the five groups included four animals: **A**. One week group, occipital cortex and leptomeningeal vessels were HIF-1α (+ + +) (HIF-1α staining , 10 × 10); **B**. Two weeks group, occipital cortex and soft meningeal blood vessels showed HIF-1α (+) (HIF-1α staining , 10 × 10); **C**. three weeks group, pial vessels in occipital cortex had HIF-1α (±) (HIF-1α staining , 10 × 10); **D**. control group, occipital cortex and leptomeningeal vessels were HIF-1α (±) (HIF-1α staining , 10 × 10); **E**. 90 day group, dura was HIF-1α (±), the number of visible blood vessels increased (HIF-1α staining , 10 × 10).

VEGF expression in the occipital cortex and vessels in the pia mater originating from the occipital lobe in the 1 week and 2 weeks groups were significantly higher than in control group, 3 weeks, and 90-days groups (p ≤ 0.002). Compared with the animals in control group, the SSS of the animals in the 2 weeks groups showed significantly higher VEGF expression level (p = 0.002). Compared with the control group and the 2 weeks, 3 weeks, and 90-days groups, the one week group had shown higher HIF-1α expression in the occipital cortex, vessels in the pia mater and SSS (p ≤ 0.002; Table 2). VEGF and HIF-1α expression levels by immunohistochemistry were depicted in Figures 6 and 7, respectively.

**Table 2:** **Immunohistochemistry results for VEGF and HIF-1α expression level**

		Groups					P-value
		Control (n = 6)	1-week (n = 6)	2-weeks (n = 6)	3-weeks (n = 6)	90-days (n = 6)	
VEGF expression level in occipital cortex*	-	1	0	0	1	1	<0.001
	±	5	0	0	5	5	
	+	0	6	1	0	0	
	++	0	0	5	0	0	
VEGF expression level in vessels in pia mater*	-	2	0	0	1	2	<0.001
	±	4	0	0	5	4	
	+	0	6	2	0	0	
	++	0	0	4	0	0	
VEGF expression level in superior sagittal sinus†	-	1	0	0	0	1	0.004
	±	5	4	0	2	0	
	+	0	2	6	4	5	
	++	0	0	0	0	0	
VEGF expression level in vessels in dura mater	-	1	0	0	0	1	0.002
	±	5	5	1	1	0	
	+	0	1	5	5	5	
	++	0	0	0	0	0	
HIF-1α expression level in cortex of occipital lobe‡	-	2	0	0	1	0	<0.001
	±	4	0	1	5	6	
	+	0	0	5	0	0	
	++	0	2	0	0	0	
	+++	0	4	0	0	0	
HIF-1α expression level in vessels in pia mater‡	-	0	0	0	2	1	<0.001
	±	6	0	1	4	5	
	+	0	0	5	0	0	
	++	0	1	0	0	0	
	+++	0	5	0	0	0	
HIF-1α expression level in superior sagittal sinus‡	-	2	0	0	0	1	<0.001
	±	4	0	1	1	5	
	+	0	0	5	5	0	
	++	0	2	0	0	0	
	+++	0	4	0	0	0	
HIF-1α expression level in dura mater‡	-	2	0	0	0	1	<0.001
	±	4	0	1	2	5	
	+	0	0	5	4	0	
	++	0	2	0	0	0	
	+++	0	4	0	0	0	

*Higher expression levels were observed in the 1 week and 2 weeks groups compared to control group, 3 weeks, and 90 days groups. The percentage was expressed as stained “positive” cell divided by the total number of cells under the same magnifications (i.e., 100× or 200×) of the same visual areas. The criteria used to assign the numerical values to expression levels were the following: - = 0%, + = >0%-25%, ++ = 26%-50%, +++ = 51%-75%, ++++ = >75%. †Higher expression level was observed in the 2 weeks group compared to control group. ‡Higher expression levels were observed in the one week group compared to control group, 2 weeks, 3 weeks, and 90 days groups.

### VEGF and HIF-1α expression levels by Western blot analysis

**Figure 8:**
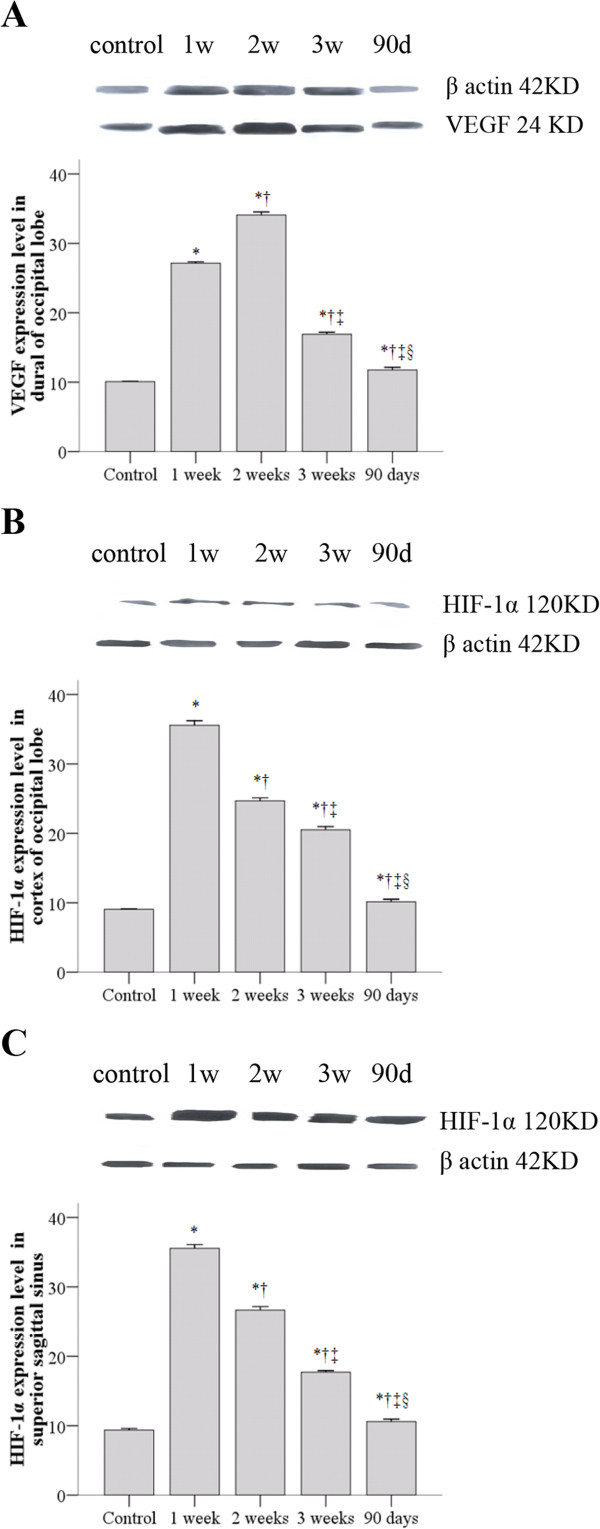
**VEGF and HIF-1α expression levels by Western blot analysis.** Each of the five groups included four animals. Specimens were collected from the control, one week, two weeks, three weeks, and 90 days groups, respectively. **A**. VEGF expression level in dural of occipital lobe; **B**. HIF-1α expression level in cortex of occipital lobe; **C**. HIF-1α expression level in superior sagittal sinus. *Statistically significant compared to control group. †Statistically significant compared to 1 week group. ‡ Statistically significant compared to 2 weeks group. § Statistically significant compared to 3 weeks group.

The patterns of VEGF expression level in the dura of the occipital lobe as well as HIF-1α expression level in the cortex of the occipital lobe and SSS were similar. The peak expression levels were observed in the 1 week group, followed by the 2 weeks, 3 weeks, 90 days and control groups. The mean expression levels for each corresponding control, 1 week, 2 weeks, 3 weeks, and 90 days were 10.1, 27.2, 34.1, 16.9, and 11.8 for VEGF expression in the dura of the occipital lobe, respectively; 9.1, 35.6, 24.7, 20.5, 10.1 for HIF-1α expression in the cortex of the occipital lobe, respectively, and 9.4, 35.6, 26.7, 17.7, 10.6 for HIF-1α expression level in the SSS, respectively. All comparisons between each two groups were statistically significant (Figure 8).

## Discussion

In this study, using a rabbit model, we showed that DAVF formation could be produced by EEA and ESA of the right side CCA-PFV + ligation of the contralateral EJV. Our results indicated that the mechanism involved anatomosis generating high intracranial venous pressure which caused ischemia and hypoxia which increased the expression of HIF-1α and subsequently VEGF in the occipital cortex, pial vessels from the same occipital cortex, and the dura on SSS after surgery. This is the first report of a rabbit model using CCA-PFV anastomosis to generate high intracranial venous pressure and successfully induce DAVP formation. It also may be the first report of a study using this rabbit model in which HIF-1α and VEGF were expressed in sequential order. The clinical implications of our findings are that understanding the details of the mechanisms of DAVF formation may permit the development of better therapeutic strategies for treating this condition.

### Mechanism of DAVF formation

Intracranial high venous pressure appears to the key to DAVF formation. High venous pressure may result from venous sinus thrombosis or congenital thrombosis. There are various opinions about how high venous sinus pressure induces DAVF and many studies have been carried out that have focused on this issue. Sandalcioglu et al. found that the plasma VEGF level was significantly higher in patients with cerebral vascular malformations compared with healthy people [7]. According to this result, they presumed that VEGF may play a special role in cerebral vascular malformations. Kim et al. analyzed the plasma VEGF level in 13 patients with cerebral malformations before and after treatment and found that the plasma VEGF level was significantly lower before surgery in these patients compared with healthy people, and further decreased within 24 h after surgery but became normal by 30 days after surgery [5]. Therefore, they suggested that abnormal vascular proliferation may play an important role in cerebral arteriovenous malformations. Although in both studies it was concluded that VEGF may play an important role in cerebral arteriovenous malformations, the changes of the plasma VEGF level were directly opposite in these two studies. Therefore, the relationship between VEGF and cerebral arteriovenous malformation needs further clarification.

### Role of vascular growth factors

Many investigators have studied the pathology of dura removed from DAVF patients and found that there were changes of various vascular growth factors. Tirakotai et al. found that the rates of VEGF and HIF-1α expression in the dura of DAVF patients were 100% and the rates of transforming growth factor α (TGFα) and basic fibroblast growth factor (bFGF) were 75% [8]. Increased VEGF, TGFα and bFGF expression mainly occurred in the endothelial cell layer. Uranishi et al. compared the dural VEGF and bFGF expression in patients with DAVF and associated venous sinus thrombosis and in patients who died of non-central nervous system diseases and found that the VEGF and bFGF expression levels were relatively low in the control group; the main locations of VEGF and bFGF expression were the subendothelial layer, middle vascular layer and perivascular tissue [9]. In the DAVF group, the locations were the same with regard to expression level, but bFGF expression was stronger and the concentration of staining was higher. In addition, the bFGF expression could be observed in the arterioles on the wall of the dural sinus. VEGF expression was observed in the wall of the diseased dural sinus and the arterioles with organized thrombus in the venous sinus. Tirakota et al. found that the dural proliferating cell nuclear antigen (PCNA), VEGF, ephrin-B2 and Fik-1 and Fit-1 (VEGF receptors) expression levels were 50%, 100%, 86.7% , 33.3% and 66.7% higher, respectively, in DAVF patients [10]. VEGF and ephrin-B2 expression were mainly located in the endothelial cell layer. Investigators have noted that it is obvious that DAVF patients have obvious changes of the vascular growth factors in the endothelial cells [9, 11] and the peripheral tissues [9]. Li et al. used rats to investigate whether the VEGF signaling pathway has an important role in DAVF development found that VEGF and its receptor may play have such a role in DAVF induced by venous hypertension [12].

The vascular remodeling (expression of endothelial growth factor and angiogenesis) due to vascular lesion-induced high venous pressure and hypoxia [8, 10] or hemodynamic changes [9] has been proposed to be the key factor for new DAVF formation. Zhu et al. exposed rats to venous hypertension for various periods of time and found that compared with controls there was a significant increase in the expression of hypoxia inducible factor-1α (HIF-1α) [10]. There was also a significant increase in the expression of vascular endothelial growth factor (VEGF). The authors concluded that angiogenesis was stimulated by venous hypertension through a mechanism that did not involve ischemia. Shin et al. used three DAVF rat models and found that the model that employed CCA-EJV anastomosis and bipolar coagulation of the vein that drains the transverse sinus and sagittal sinus thrombosis to induce venous hypertension had the highest level of VEGF, and in this model VEGF peaked one week following surgery thus appearing to demonstrate that an angiogenic growth factor contributed to DAVF formation [13].

### High venous sinus pressure

Many researchers have established rat models of high venous sinus pressure using CCA-PFV anastomosis method and found that (1) the high venous sinus pressure may promote the expression of vascular growth factors [14]; (2) the high venous sinus pressure may induce DAVF [8]; (3) strong VEGF expression could be observed in the astrocytes [7], venous endothelial cells, connective tissues around the venous sinus, dural endothelial layer of the dura, cerebral cortex and basal ganglia [15], etc.; and (4) there was a certain correlation between the extent of VEGF expression and the level of pressure as VEGF expression increased along with the increase of the pressure.

**Figure 9:**
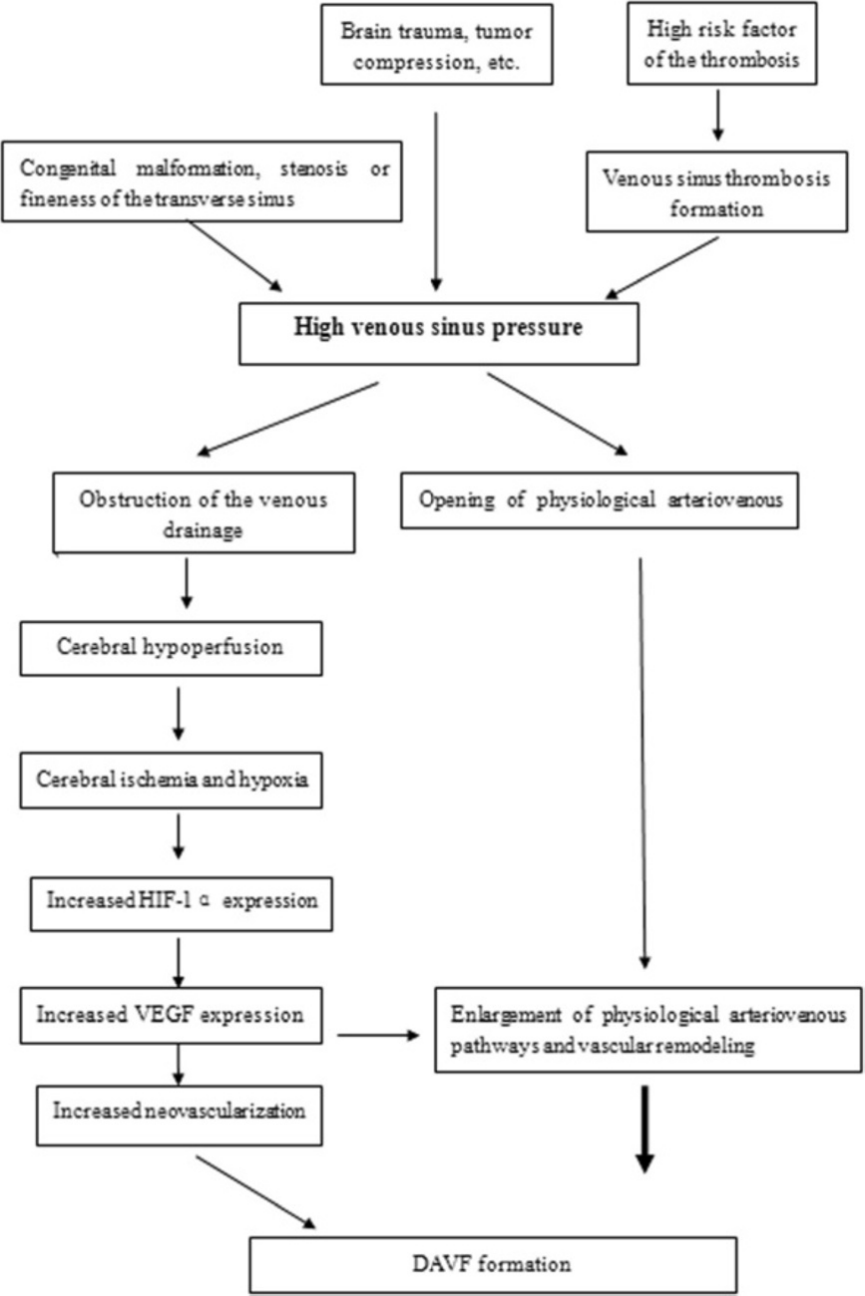
**Flow diagram illustrating a proposed mechanism for explaining how high venous sinus pressure can result in DAVF formation.**

We believe that high intracranial pressure is the key factor in DAVF formation. There are “direct physiological arteriovenous pathways” in the dural vascular structures. These physiological pathways are open when there is a high venous pressure, which results in fistula formation. However, the blood flow in these fistulae is limited and cannot form a DAVF immediately; further vascular remodeling is required. During the observation following dural ink perfusion in rabbits 1-3 weeks after surgery, enlargement of these direct pathways could be observed without fistula formation. The high venous sinus pressure may result in decreased cerebral perfusion pressure and induce cerebral ischemia. Hypoxia after high intracranial venous pressure leads to the following pathophysiological changes: 1) HIF expression is increased and then VEGF is activated, 2) VEGF has the highest expression in the sinus surrounding the most severe ischemia, 3) a dural venous sinus is formed and dural neovascularization is observed to increase, 4) part of the neovascularization is connected with the original artery which results in arterial blood flowing directly into the sinus resulting in DAVF formation. To adapt to the cerebral ischemia and improve the hypoxic environment, vascular growth factors including HIF-1α, VEGF, etc. are highly expressed. The long-distance arteriovenous direct pathways can significantly increase the vascular resistance and reduce the venous pressure. If the distance is short, the capillary pressure and venous pressure obviously increase and remodeling of this kind of direct pathway may occur under the effect of vascular growth factors and smooth muscle cells appear in the peripheral area to form a DAVF [16]. During the observation of rat dural specimens [17], these kind of specific pathways were most commonly seen in the dura of adjacent sinuses. Therefore, DAVF formation easily occurs nearby the sinus. We consider that the high venous sinus pressure is a “trigger point” inducing DAVF formation. The detailed processes are illustrated in Figure 9.

### Limitations

Ours study had limitations because of our rabbit model had the following shortcomings: (1) We considered that the PFV pressure could indirectly reflect the intracranial venous pressure. Because of the effect of anatomical structures there is a certain deviation in this value. (2) Because the diameter of the PFV is more than two times that of the CCA it is difficult to form the arteriovenous anastomosis. (3) We did not design an animal group with venous sinus thrombosis and the effect of thrombosis on DAVF formation was not studied, (4) DSA examination was carried out after placing a catheter in the left CCA. The examination of the intracranial blood vessels was insufficient and changes in the right side veins were not evaluated. There is no proper justification on the number of animals used for this study Even we had budget limitations, we attempted to maximize the whole experimental design to obtain as much data as possible within 100 rabbits usedin this study.

## Conclusions

The results of our rabbit model experiments indicate that high intracranial pressure plays a key role in DAVF formation. Cerebral ischemia caused by a lack of cerebral perfusion pressure plays a key role in a process that leads from high intracranial venous pressure to increased HIF-1α expression and subsequently increased VEGF expression.

## Electronic supplementary material

Additional file 1: Figure S1: **S1A.** Schematic drawings of group A (the control group). Group A. a: superior sagittal sinus (SSS); b: right-side posterior-facial vein (PFV); c: right-side anterior-facial vein (AFV); d: right-side common carotid artery (CCA); e: right-side vertebral artery (VA). **S1B**. Schematic drawings to illustrate the vessel that were occluded and anastomosed in group B and group C. Group B (right-side CCA-PFV EEA) and group C (right-side CCA-PFV EEA plus left-side EJV ligation). a: superior sagittal sinus (SSS); b: right-side posterior-facial vein (PFV); c: right-side anterior-facial vein (AFV); d: right-side common carotid artery (CCA); e: right-side vertebral artery (VA); f: residue of ligation of right common carotid artery; g: right-side CCA-PFV EEA; h: right-side EJV ligation; j: left-side EJV ligation. Arrow indicated blood stream direction. **S1C**. Schematic drawings to illustrate the vessel that were occluded and anastomosed in group D and group E. Group D: right-side CCA-PFV end-to-side anastomosis and group E right-side CCA-PFV end-to-side anastomosis plus left-side EJV ligation. a: superior sagittal sinus (SSS); b: right-side posterior-facial vein (PFV); c: right-side anterior-facial vein (AFV); d: right-side common carotid artery (CCA); e: right-side vertebral artery (VA); g: right-side CCA-PFV end-to-side anastomosis; h: right-side EJV ligation; j: left-side EJV ligation. Arrow indicated blood stream direction. (TIFF 961 KB)

## Abbreviations

AFV: Anterior-facial vein
BA: Basilar artery
bFGF: Basic fibroblast growth factor
CCA: Common carotid artery
DSA: Digital subtraction angiography
DAVF: Dural arteriovenous fistula
EA: Ear artery
EEA: End-to-end anastomosis
ESA: End-to-side anastomosis
EV: Ear vein
ECA: External carotid artery
EJV: External jugular vein
HIF-1α: Hypoxia inducible factor-1α
IV: Intravenous
ICA: Internal carotid artery
OV: Ophthalmic vein
PFV: Posterior facial vein
SSS: Superior sagittal sinus
TGFα: Transforming growth factor α
VEGF: Vascular endothelial growth factor
VA: Vertebral artery.
