# Supplementary figures and images for: Investigation of the mechanism of dural arteriovenous fistula formation induced by high intracranial venous pressure in a rabbit model
Source: BMC Neurosci. 2014 Aug 27;15:101. doi: 10.1186/1471-2202-15-101 (PMC4152575; doi:10.1186/1471-2202-15-101)

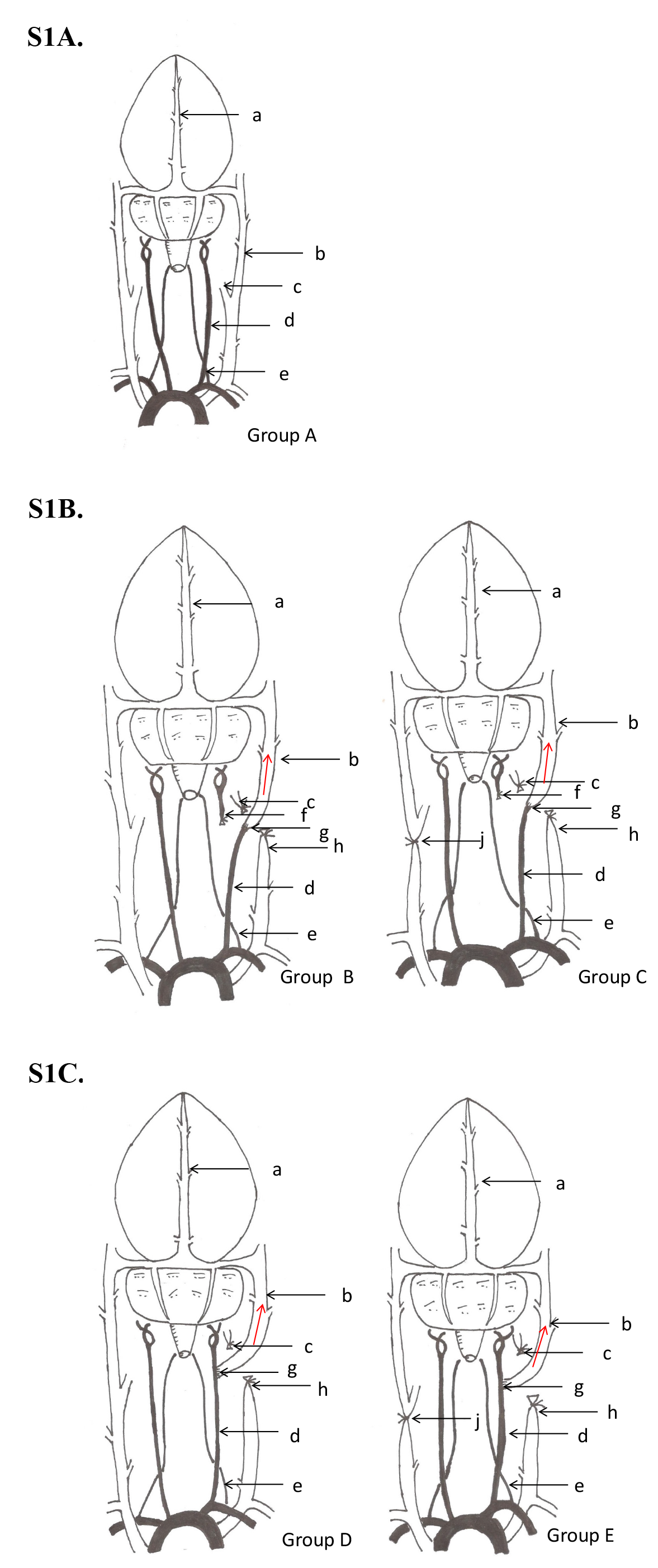

Supplement: Supplementary file 1 — Additional file 1: Figure S1: S1A. Schematic drawings of group A (the control group). Group A. a: superior sagittal sinus (SSS); b: right-side posterior-facial vein (PFV); c: right-side anterior-facial vein (AFV); d: right-side common carotid artery (CCA); e: right-side vertebral artery (VA). S1B. Schematic drawings to illustrate the vessel that were occluded and anastomosed in group B and group C. Group B (right-side CCA-PFV EEA) and group C (right-side CCA-PFV EEA plus left-side EJV ligation). a: superior sagittal sinus (SSS); b: right-side posterior-facial vein (PFV); c: right-side anterior-facial vein (AFV); d: right-side common carotid artery (CCA); e: right-side vertebral artery (VA); f: residue of ligation of right common carotid artery; g: right-side CCA-PFV EEA; h: right-side EJV ligation; j: left-side EJV ligation. Arrow indicated blood stream direction. S1C. Schematic drawings to illustrate the vessel that were occluded and anastomosed in group D and group E. Group D: right-side CCA-PFV end-to-side anastomosis and group E right-side CCA-PFV end-to-side anastomosis plus left-side EJV ligation. a: superior sagittal sinus (SSS); b: right-side posterior-facial vein (PFV); c: right-side anterior-facial vein (AFV); d: right-side common carotid artery (CCA); e: right-side vertebral artery (VA); g: right-side CCA-PFV end-to-side anastomosis; h: right-side EJV ligation; j: left-side EJV ligation. Arrow indicated blood stream direction. (TIFF 961 KB) [file 12868_2014_3788_MOESM1_ESM.tiff]
